# New data from basal Australian songbird lineages show that complex structure of MHC class II β genes has early evolutionary origins within passerines

**DOI:** 10.1186/s12862-016-0681-5

**Published:** 2016-05-21

**Authors:** Shandiya Balasubramaniam, Rebecca D. Bray, Raoul A. Mulder, Paul Sunnucks, Alexandra Pavlova, Jane Melville

**Affiliations:** Department of Sciences, Museum Victoria, Melbourne, VIC 3001 Australia; School of BioSciences, The University of Melbourne, Melbourne, VIC 3010 Australia; Terrestrial Vertebrates, Western Australian Museum, Perth, WA 6986 Australia; School of Biological Sciences, Monash University, Melbourne, VIC 3800 Australia

**Keywords:** Trans-species polymorphism, Gene duplication, Concerted evolution, Convergent evolution, Birth-and-death model, Accordion model, Passerida, Corvida

## Abstract

**Background:**

The major histocompatibility complex (MHC) plays a crucial role in the adaptive immune system and has been extensively studied across vertebrate taxa. Although the function of MHC genes appears to be conserved across taxa, there is great variation in the number and organisation of these genes. Among avian species, for instance, there are notable differences in MHC structure between passerine and non-passerine lineages: passerines typically have a high number of highly polymorphic MHC paralogs whereas non-passerines have fewer loci and lower levels of polymorphism. Although the occurrence of highly polymorphic MHC paralogs in passerines is well documented, their evolutionary origins are relatively unexplored. The majority of studies have focussed on the more derived passerine lineages and there is very little empirical information on the diversity of the MHC in basal passerine lineages. We undertook a study of MHC diversity and evolutionary relationships across seven species from four families (Climacteridae, Maluridae, Pardalotidae, Meliphagidae) that comprise a prominent component of the basal passerine lineages. We aimed to determine if highly polymorphic MHC paralogs have an early evolutionary origin within passerines or are a more derived feature of the infraorder Passerida.

**Results:**

We identified 177 alleles of the MHC class II β exon 2 in seven basal passerine species, with variation in numbers of alleles across individuals and species. Overall, we found evidence of multiple gene loci, pseudoalleles, trans-species polymorphism and high allelic diversity in these basal lineages. Phylogenetic reconstruction of avian lineages based on MHC class II β exon 2 sequences strongly supported the monophyletic grouping of basal and derived passerine species.

**Conclusions:**

Our study provides evidence of a large number of highly polymorphic MHC paralogs in seven basal passerine species, with strong similarities to the MHC described in more derived passerine lineages rather than the simpler MHC in non-passerine lineages. These findings indicate an early evolutionary origin of highly polymorphic MHC paralogs in passerines and shed light on the evolutionary forces shaping the avian MHC.

**Electronic supplementary material:**

The online version of this article (doi:10.1186/s12862-016-0681-5) contains supplementary material, which is available to authorized users.

## Background

The major histocompatibility complex (MHC) is a complex multigene family that regulates the function of the adaptive branch of the vertebrate immune system. The MHC is comprised of many different types of genes, such as classical and non-classical MHC genes, as well as non-MHC genes such as natural killer cells and tapasin [1]. Classical and non-classical MHC genes may be distinguished from each other in that the former are polymorphic and highly expressed, whereas the latter exhibit lower levels of expression and are often monomorphic [2]. MHC genes may also be grouped as class I, II or III genes based on the types of molecules they encode. Class I genes encode receptors that are presented on the surfaces of most nucleated cells and primarily facilitate immune responses to intracellular pathogens, whereas class II genes are only found on a subset of cells and are associated with immune responses to extracellular pathogens [3]. Class III genes encode molecules involved in the complement component of the immune response rather than the adaptive response [2].

The high levels of polymorphism characteristic of classical MHC class I and II genes may be largely attributed to the peptide-binding region (PBR), which displays great diversity of alleles and extensive sequence variation among different alleles [3, 4]. The genes of this region encode proteins that form the molecular groove where peptides are bound and then presented to T-cells, resulting in the appropriate immune response being triggered [1]. Each allele typically responds to a category of potential antigens, so individuals and populations with greater MHC diversity may be better able to cope with a range of infections [5, 6].

A number of models of multigene family evolution, such as the birth-and-death model [7] and the accordion model [5], have been proposed to explain the generally high levels of polymorphism and the large and variable number of classical MHC loci among individuals and species. According to the birth-and-death model, new genes are created via repeated duplication (birth) and then either retained in the genome or eventually lost (death) after becoming non-functional through deleterious mutations [7]. The accordion model, on the other hand, posits that the number of MHC genes expands and contracts in response to fluctuating selection pressure [5]. These models are not mutually exclusive, and both models predict the redundancy that occurs in the genome of most species, whereby multiple duplicated gene loci exist in both class I and II gene regions [6]. One explanation for this redundancy is that once alleles are generated, they may remain in the genome even in the absence of selection pressure [6]. Alleles may be maintained over long evolutionary time scales through speciation events, which may then resolve as trans-species polymorphisms (TSP) in phylogenetic reconstructions, where similar alleles are present in groups that diverged over millions of years ago such as rodents and primates [10, 11]. On a smaller scale, genetic variation in the MHC may also be generated among alleles and between loci via the processes of point mutation, recombination and gene conversion, and maintained through the influence of balancing selection.

Among birds, the most studied and well-described MHC is that of the chicken (*Gallus gallus*), which only contains one dominantly expressed molecule of class I and one of class II [6, 7]. The chicken MHC is small and densely arranged; a compact organisation of genes with short introns, small physical size, lack of redundancy, and low overall number of classical class I and II genes with few pseudogenes, has led to the chicken MHC being described as a “minimal essential MHC” [7, 8]. The small size and simplicity of the chicken MHC allows for the co-evolution of genes as haplotypes over considerable periods of time, with no recombination between class I and II genes having been detected over thousands of experiments [7, 9]. The result of this is that the most striking associations between MHC haplotype and resistance or susceptibility to disease have been characterised in chickens [10, 11].

Similarly simple genetic organisation and small numbers of expressed classical MHC loci have been noted in a few galliform species, such as the pheasant (*Phasianus colchicus*) [12], black grouse (*Tetrao tetrix*) [23], and grey partridge (*Perdix perdix*) [13], although the quail (*Coturnix japonica*) has been found to have higher levels of gene duplication [14]. Variable levels of MHC complexity have been characterised among other avian groups, such as penguins, seabirds, raptors, rails, cranes, and waders [15–20], and passerine species typically show multiple loci with extensive gene duplication, evidence of recombination, high levels of polymorphism, and the presence of pseudogenes [21–25]. This suggests the minimal essential model is not applicable to all avian species, as the passerine MHC appears to be larger and more complex than that described in chickens. The passerine MHC, along with a number of non-passerine lineages, has been characterised as having high rates of concerted evolution and/or recent duplications [2, 12], leading to few reports of orthologous relationships among birds [12, 26–29]. Thus, although the overall function of the MHC is conserved across birds, there appear to be considerable differences in the genomic organisation of the MHC between lineages [30].

**Fig. 1:**
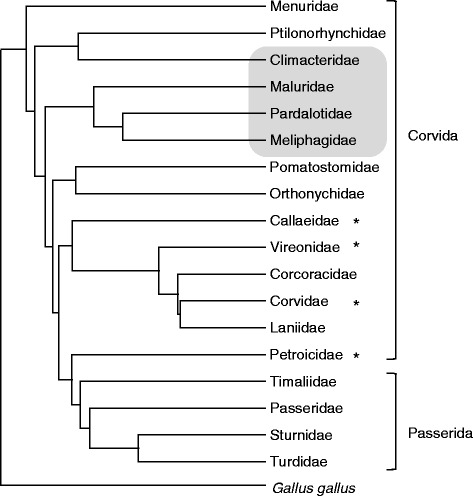
Relationships among major passerine families. Grey shading indicates families included in this study and asterisks denote families in the Corvida that have been previously studied. Phylogeny constructed using the Ericson backbone in birdtree.org [72] and rooted with *Gallus gallus*

With a few exceptions [27, 31–33], research on the MHC in passerines has focussed on species within the more derived infraorder Passerida, whereas very little empirical information exists on the structure and complexity of the MHC in the Corvida (*sensu* [34]), a paraphyletic basal clade within the passerines (Fig. 1). It remains unclear whether a complex MHC is characteristic of all passerines or is a more derived trait occurring in the Passerida and the few species of the Corvida that have been studied (Fig. 1). Here, we used cloning and next-generation 454 sequencing techniques to characterise the polymorphism and allelic diversity of classical MHC class II β exon 2. We focussed on seven Australian species from four prominent basal passerine families (Table 1, Fig. 1) to test if there is increasing complexity of the MHC structure from basal to derived passerines: if there is such a progressive increase in MHC complexity, basal passerine lineages should show a more simple MHC structure similar to that described in non-passerine lineages. To better understand the evolutionary origins of complex MHC structure in passerines, we also reconstructed phylogenetic relationships among MHC sequences in these species.

**Table 1:** Sample information and MHC allelic diversity

Family	Species	Species prefix	N_A_ (range)	N_loci_	N_ps_
Climacteridae	Brown treecreeper *Climacteris picumnus*	Clpi	30 (8–17)	9	0
Melphagidae	Yellow-tufted honeyeater *Lichenostomus melanops*	Lime	39 (11–14)	7	2
	White-plumed honeyeater *Lichenostomus penicillatus*	Lipe	19 (6–11)	6	0
	Fuscous honeyeater *Lichenostomus fuscus*	Lifu	24 (6–10)	5	1
Maluridae	Superb fairy-wren *Malurus cyaneus*	Macy	33 (2–14)	7	2
Pardalotidae	Spotted pardalote *Pardalotus punctatus*	Papu	26 (5–11)	6	3
	Striated pardalote *Pardalotus striatus*	Past	39 (7–13)	7	1

N_A_ - total number of alleles per species; range - range of alleles detected in individuals of each species; N_loci_ – putative number of loci per species; Nps - number of pseudoalleles

## Results and discussion

In order to assess levels of polymorphism and allelic diversity in the MHC class II genes of basal passerines, we chose the following species: Brown Treecreeper *Climacteris picumnus*, Superb Fairy-wren *Malurus cyaneus*¸ Spotted Pardalote *Pardalotus punctatus*, Striated Pardalote *P. striatus*, White-plumed honeyeater *Lichenostomus penicillatus*, Fuscous honeyeater *L. fuscus*, and Yellow-tufted Honeyeater *L. melanops*. These seven species are from four families (Climacteridae, Maluridae, Pardalotidae, Meliphagidae) that comprise a prominent component of the Corvida. We amplified a 159 bp region that includes the highly diverse PBR of the MHC class II β exon 2 using cloning and 454 sequencing methods. The high level of coverage provided by 454 sequencing makes it a convenient method for assessing genetic diversity in highly polymorphic, multilocus systems, such as the MHC [25, 35, 36]. We used 454 sequencing and cloning with Sanger sequencing complementarily to assess levels of genetic diversity in seven basal passerine species.

The two methods resulted in different numbers of alleles being identified in each species. We isolated 98 MHC class II β exon 2 sequences from 21 individuals using cloning with Sanger sequencing (see Additional file 1), with the total number of alleles detected in each species ranging from 7 in the Spotted Pardalote to 19 in the Striated Pardalote. Of the 98 sequences isolated, 17 were found in more than one species, resulting in 81 unique alleles across the seven species. Sample sizes were the same in every species and all samples were from the same study region, so the variation in allele numbers across species is unlikely to be a product of sampling design. Within species, the number of alleles identified in each individual ranged from 5 to 14.

454 sequencing was conducted on 13 samples in total (see Additional file 1), across all seven study species. Due to small volumes of available blood samples, different individuals were sequenced using 454 compared to Sanger sequencing, and from a different location in the study area (see Methods). Using 454 sequencing, we obtained 16,257 reads of the 159-bp region showing a complete match to the forward and reverse primers. After a stepwise filtering procedure, we were left with 66 % of the original reads (10,783), with coverage depth ranging from 114 to 570 reads per allele (see Additional file 2). This remaining 66 % of reads comprised a total of 146 alleles, of which 12 were found in more than one species. Of the remaining 134 unique alleles recovered through 454 sequencing, 38 were also identified through cloning. Similar to the sequences isolated through cloning, numbers of alleles varied widely across species and individuals, ranging from 11 in the White-plumed Honeyeater to 26 in the Brown Treecreeper. The isolation of more than two alleles from each individual indicates the presence of multiple MHC class II β loci in each of these species, from a minimum of five loci in the Fuscous Honeyeater to nine loci in the Brown Treecreeper. The conservative filtering process we used would have potentially excluded a larger number of true alleles from individuals with higher coverage compared to individuals with lower coverage, so levels of MHC allelic diversity may be underestimated in some species.

Despite fewer individuals having been screened through 454 sequencing compared to cloning, larger numbers of alleles were identified in total and in each individual (see Additional file 1). Although 454 sequencing has the advantage of avoiding some artefacts associated with bacterial cloning, it is still vulnerable to artefacts arising during PCR and has been shown to produce a higher percentage of sequencing errors than Sanger sequencing [37–40]. In contrast, Sanger sequencing probably represents a smaller proportion of the allelic diversity of a species, compared with 454 sequencing, with a prevalence of locally common alleles while less common (or rare) alleles are missed in cloning. Our use of both these methods suggests 454 sequencing is a much more efficient, although possibly less accurate, way to assess levels of allelic diversity in species and populations.

### Polymorphism and allelic diversity of MHC loci

We identified a total of 177 alleles of the MHC class II β exon 2 across seven species. Numbers of alleles varied widely across species and individuals, from 19 to 39 alleles per species and 2 to 17 alleles per individual (Table 1). Based on the maximum number of alleles in an individual, we inferred a minimum of 5–9 loci per species, a range which falls within the middle of the spectrum for the number of MHC class II β loci described in passerines (*N* = 3–20; [22]). Frameshift mutations and stop codons in sequences from five of the seven species suggest pseudoalleles are present in the dataset (Table 1), a level similar to that found in the infraorder Passerida [41, 42]. All seven species displayed high levels of allelic diversity, with high intraspecific nucleotide and amino acid distances compared to other passerines ([43, 44]; Table 2). The highest levels of diversity were observed at the PBR in all species, a pattern again consistent with Passerida MHC [2].

**Table 2:** Mean nucleotide and amino acid distances

	Nucleotide			Amino acid		
Species	All sites	PBR	non-PBR	All sites	PBR	non-PBR
Clpi	0.147 (0.019)	0.266 (0.054)	0.110 (0.021)	0.245 (0.046)	0.574 (0.125)	0.154 (0.045)
Lime	0.229 (0.023)	0.455 (0.072)	0.167 (0.022)	0.423 (0.062)	0.845 (0.202)	0.315 (0.059)
Lipe	0.253 (0.026)	0.513 (0.080)	0.184 (0.025)	0.409 (0.064)	0.890 (0.193)	0.288 (0.058)
Lifu	0.259 (0.025)	0.539 (0.072)	0.188 (0.025)	0.421 (0.062)	0.909 (0.183)	0.302 (0.059)
Macy	0.318 (0.028)	0.521 (0.078)	0.263 (0.030)	0.536 (0.071)	0.939 (0.197)	0.433 (0.071)
Papu	0.134 (0.018)	0.332 (0.068)	0.077 (0.014)	0.229 (0.046)	0.624 (0.158)	0.123 (0.034)
Past	0.141 (0.019)	0.338 (0.064)	0.084 (0.015)	0.246 (0.048)	0.641 (0.166)	0.141 (0.036)

Mean nucleotide and amino acid distances among putative alleles from MHC class II β exon 2. Nucleotide distances are corrected for multiple substitutions with the Kimura 2-parameter model and amino acid distances are corrected using expectations from the Poisson distribution. Standard errors are based on 1000 bootstrap replicates and given in parentheses. *PBR* peptide binding region

### Phylogenetic relationships among MHC alleles

**Fig. 2:**
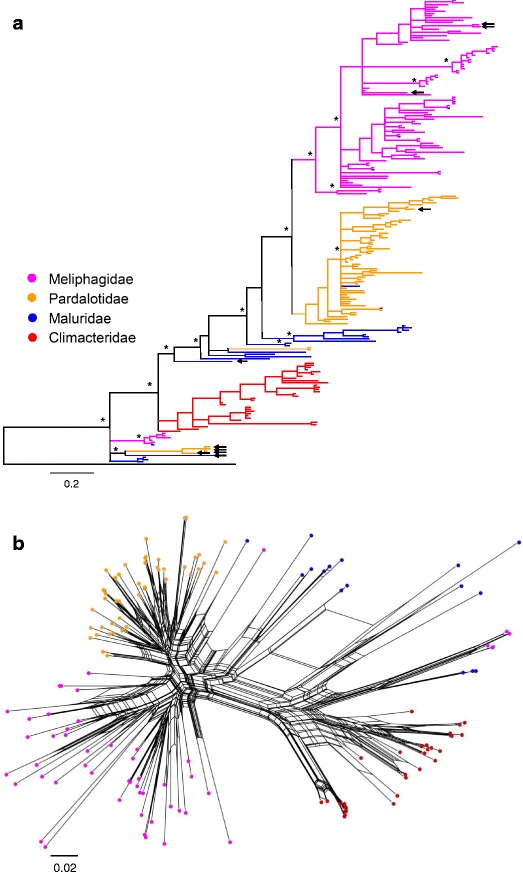
Relationships of MHC class II β exon 2 sequences among basal passerine species. **a** Bayesian phylogeny of MHC alleles from the seven basal passerine species in this study, rooted with *Crocodylus niloticus*; asterisks denote major branches with posterior probabilities > 90 %, and pseudoalleles are indicated by arrows. **b** Neighbour-net of MHC class II β exon 2 sequences from seven basal passerine species. Sequences are colour coded by family

Bayesian reconstruction of the phylogenetic relationships among MHC class II β exon 2 alleles from the seven species largely reflected taxonomic relationships at the genus level. The majority of alleles clustered in one of three well-supported clades (≥90 % posterior probability; Fig. 2), with alleles from honeyeaters (Meliphagidae), pardalotes (Pardalotidae), and treecreepers (Climacteridae) grouping separately to each other. The phylogenetic network showed a similar pattern of clustering, with alleles from honeyeaters, pardalotes and treecreepers falling into three distinct clusters (Fig. 2). Sequences from the Superb Fairy-wren (Maluridae) were the exception to this general pattern, where approximately half of the sequences (15/33 sequences; 45 %) were basal to the main Pardalotidae-Meliphagidae lineage in the Bayesian tree but did not form a monophyletic clade, and other Maluridae sequences were intermingled within Climacteridae, Pardalotidae and Meliphagidae.

The basal position of the Superb Fairy-wren sequences may result from recombination generating a pattern of mixed ancestry, which causes recombinant sequences to fall outside the parental clusters [45]. The intermingling of other Superb Fairy-wren sequences within clades in the phylogenetic tree containing other genera may indicate a trans-species mode of evolution, a pattern which has been widely documented in MHC class II β sequences of both passerines and non-passerines [28, 46–50]. Trans-species polymorphism (TSP) is a mechanism by which identical alleles occur in related species by being passed on from ancestral to descendant species [51]. This can be difficult to differentiate from convergent evolution, which can produce similar alleles in different species through independent evolutionary pathways. Being sympatric, the seven species in this study may display signatures of convergent evolution if they are subject to similar parasite or pathogen pressure. To differentiate between signatures of convergent evolution and TSP, we compared phylogenetic reconstructions based on non-synonymous and synonymous substitutions only. If the species are subject to similar selective pressures, the phylogeny based on non-synonymous substitutions should show mixed clustering of sequences across families whereas the phylogeny based on synonymous substitutions should show alleles grouping according to family or species. If, however, the similarity of alleles across species is a result of TSP, the two phylogenies should show similar patterns of allelic clustering across families [48, 52]. A comparison of phylogenies based on non-synonymous and synonymous substitutions showed the latter pattern of clustering, with the majority of clusters in both phylogenies showing alleles from more than one family (see Additional file 3). Mixed clusters predominantly comprised alleles from honeyeaters, pardalotes and fairy-wrens, whereas alleles from treecreepers clustered intraspecifically for the most part. Based on our analysis of exon 2, the phylogenetic signatures support a TSP explanation of the clustering pattern rather than one of convergent evolution. Although this result suggests the presence of TSP, it must be noted that distinguishing between phylogenetic patterns generated by TSP and other non-TSP patterns and processes is problematic when the loci are unknown. The evolution of exon 2 is complex and it is possible that, for example, rates of allele sharing based only on this region are an overestimation, thereby leading to incorrect inferences of TSP [52, 53]. Comparison of phylogenies based on intron and exon regions, as well as other related species with allopatric distributions, and identification of alleles associated with individual loci, would clarify the roles of convergent evolution, TSP, and other processes in generating these patterns of clustering.

Fifteen alleles in the dataset occurred in at least two different species, and there was variation among genera in levels of TSP. In 11 instances, alleles occurred across genera between fairy-wrens and another species. In all other instances TSP occurred within genera, either between the two species of pardalotes or three species of honeyeaters. Treecreepers did not show any instances of TSP with other study species, which may be related to treecreepers being in a separate evolutionary lineage to the other study species (Climacteridae; Fig. 1). Retention of alleles through TSP between treecreepers and the other basal lineages in this study would therefore need to occur over longer evolutionary timeframes of at least 60 million years [54].

Inspection of amino acid alignments revealed seven sites with residues unique to each of the Climacteridae, Pardalotidae and Meliphagidae, distinguishing sequences in the three families from each other (see Additional file 4). Fairy-wren sequences, being intermingled within these groups, showed the residues particular to whichever group they fell in with. This pattern of clustering by species is not uncommon in birds and could be explained by either a) recent duplication, where genes diverge following speciation and then duplicate to form similar copies of the gene, or b) concerted evolution, where duplication occurs prior to speciation but the duplicated genes become homogenised through gene conversion [2]. Both these processes result in intraspecific loci being more similar to each other than to orthologous loci in other species. Among birds, patterns of orthology may be obscured because of rapid gene duplication and homogenisation of MHC class II genes [33].

**Fig. 3:**
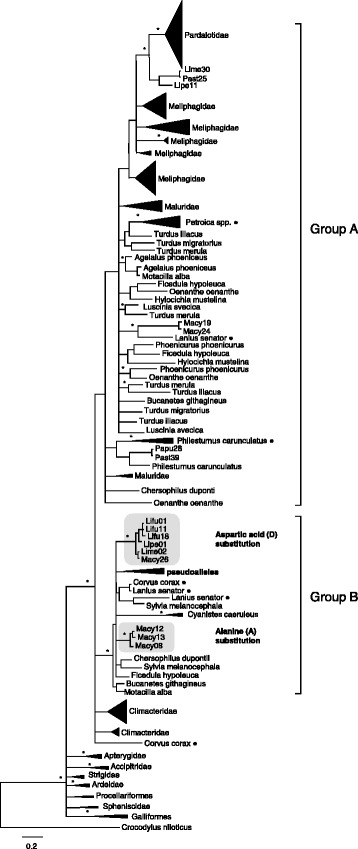
Basal passerine MHC class II β exon 2 sequences in the wider avian context. Bayesian phylogeny of MHC alleles from passerine and non-passerine families, rooted with *Crocodylus niloticus*. Asterisks denote major branches with posterior probabilities > 90 % and filled circles denote passerine species from the Corvida. Group A contains the majority of sequences from honeyeaters, pardalotes and fairy-wrens. Group B contains the remaining 18 sequences from these species, which comprise nine pseudoalleles and nine sequences with aspartic acid (D) or alanine (A) instead of glutamic acid (E) in the first position (see Additional file 4)

To assess the relationships among MHC class II β exon 2 sequences in the wider phylogenetic context, we constructed a phylogeny including species from a range of avian taxa (Fig. 3). All passerine species formed a well-supported monophyletic clade (100 % posterior probability) that was also strongly supported by topology testing, using a Bayesian stepping stone approach (Bayes Factor 75.35). All species from the non-passerine taxa (Accipitridae, Apterygidae, Ardeidae, Galliformes, Procellariformes, Spheniscidae and Strigidae) formed a polytomy at the base of the phylogeny. Within the passerine clade, most sequences from honeyeaters, pardalotes and fairy-wrens grouped together, albeit in a clade without strong posterior probability support (Group A), whereas treecreeper sequences fell separately to these sequences. The exceptions to this were one group of pseudoalleles and two groups of sequences that had either aspartic acid (D) or alanine (A) instead of glutamic acid (E) in the first position (see Additional file 4); these three groups of sequences instead clustered separately from the other sequences in this study in a well-supported clade (Group B). The clustering of pseudoalleles and putatively functional sequences (Group B) may either indicate the latter are actually non-functional alleles or that the pseudoalleles evolved from these alleles. There was no apparent structuring according to passerine taxonomic relationships, because Corvida and Passerida sequences were intermingled in the passerine clade. There is some evidence that rates of diversifying and homogenising forces may vary between lineages of birds [28], which could explain the pattern here where some sequences cluster according to species whereas other clusters comprise sequences from different species. The grouping of basal passerine species from this study with species from both the Corvida and Passerida suggests a complex MHC class II β structure may be common throughout the passerine order.

## Conclusions

In this study we detected evidence of multiple gene loci, high levels of polymorphism and allelic diversity, and the presence of pseudogenes among classical MHC class II β exon 2 of seven passerine species. Our analyses strongly support a monophyletic grouping of passerines from the Corvida and Passerida, signifying an early evolutionary origin of complex MHC class II β structure in the Passeriformes. Phylogenetic analyses based on non-synonymous and synonymous substitutions supported a trans-species explanation for the mixed clustering of alleles across the different families in this study. However, we cannot rule out the possibility that convergent evolution may have also played a role in generating these phylogenetic signatures. Phylogenetic analyses based on sequences from other MHC class II exon and intron regions may clarify the relative roles of TSP and convergent evolution in generating these signatures. A comparison of 454 sequencing and cloning methods suggested the former is a much more efficient way to assess levels of allelic diversity in species and populations. Continued characterisation of this gene region, as well as other non-coding regions of the MHC, in species from different phylogenetic levels may improve our understanding of the rates of gene conversion, recombination, diversification and homogenisation occurring in the avian MHC. Such research will also provide insights into the evolutionary significance of the disparity in MHC complexity between passerine and non-passerine species.

## Methods

### Sample collection and study design

We collected blood samples from one to three individuals of seven Australian passerine species from Victoria, Australia, as described in Amos et al. ([55]; see Additional file 1). These seven species (Brown Treecreeper *Climacteris picumnus*, Superb Fairy-wren *Malurus cyaneus*, Spotted Pardalote *Pardalotus punctatus*, Striated Pardalote *P. striatus*, White-plumed honeyeater *Lichenostomus penicillatus*, Fuscous honeyeater *L. fuscus*, Yellow-tufted Honeyeater *L. melanops*) are from four families (Climacteridae, Maluridae, Pardalotidae, Meliphagidae) that comprise a prominent component of basal passerine lineages.

As part of a pilot study we tested previously published MHC class II β primers on the study species (Additional file 5), from which we identified a single set of primers that consistently amplified across all the study species. These primers amplified a 159 bp region of the MHC class II β exon 2 and were used for all our genetic work. To assess levels of polymorphism and allelic diversity in the seven study species, we amplified the 159 bp region of the MHC class II β exon 2 using cloning and 454 sequencing methods. This 159 bp fragment includes the highly diverse PBR as well as fit within the length restrictions for 454 sequencing. The PBR and non-PBR were inferred assuming functional congruence to the human HLA-DR1 molecule [56].

### DNA extraction, PCR, cloning, and sequencing

Blood samples were digested overnight using Proteinase K and DNA was extracted with standard phenol-chloroform protocols for Sanger sequencing [57], while DNA was extracted from blood samples using a salting-out protocol for 454 sequencing [58]. The resulting DNA was suspended in Tris-EDTA. For Sanger sequencing the degenerate primers 326 and 325 [59] were used to amplify a 159 bp region of the MHC class II β gene, spanning the majority of exon 2. For 454 sequencing we amplified the same 159 bp region of the MHC class II β gene using HPLC-purified fusion primers. The forward fusion primer (5′-CCATCTCATCCCTGCGTGTCTCCGACTCAGNNNNNNNNNN**GAGTGYCAYTAYYTNAAYGGYAC**-3′) comprised the 454 GS FLX Titanium Primer A, a 10 bp Multiplex Identifier (MID) tag (indicated with Ns) to differentiate among individuals, and the 326 primer sequence (in bold). The reverse fusion primer (5′-CCTATCCCCTGTGTGCCTTGGCAGTCTCAGNNNNNNNNNN**GTAGTTGTGNCKGCAGTANSTGTCCAC**-3′) similarly comprised the 454 GS FLX Titanium Primer B, a 10 bp MID tag (indicated with Ns) and the 325 primer sequence (in bold).

For both Sanger and 454 sequencing standard 25 μl PCRs were conducted with approximately 25 ng of genomic DNA. Sanger sequencing PCRs: 1 x Taq buffer (Promega), 2.5 mM MgCl_2_, 0.2 mM of each dNTP, 0.5 units platinum *Taq* polymerase (Invitrogen), 0.02 mg/ml BSA (Sigma-Aldrich) and 0.4 mM of each primer. 454 sequencing PCRs: 1 x GoTaq Colourless Master Mix (Promega), 2 mM MgCl_2_, and 0.4 mM of each primer. A negative control was included in each PCR and a touch-down protocol was used for all amplifications. The thermal profile consisted of an initial 5 min denaturation at 95 °C, followed by 10 cycles denaturation at 95 °C for 30 s, annealing at 60–44 °C for 30 s, with the annealing temperature decreasing by 4 °C every 2 cycles, and extension at 72 °C for 90 s. This was followed by 35 cycles of denaturation at 95 °C for 45 s, annealing at 45 °C for 45 s, and extension at 72 °C for 90 s, followed by a final extension at 72 °C for 10 min. PCR products were visualised on 1.2 % agarose gels to confirm amplification.

Cloning of PCR products for Sanger sequencing was undertaken by ligating PCR product into the pCR®II-TOPO bacterial plasmid (TOPO TA Cloning® Kit Dual Promoter, Invitrogen) and transformed into TOP10F’ chemically competent cells following the manufacturer’s protocol for the TOPO TA Cloning® Kit Dual Promoter (Invitrogen). Recombinant clones were detected by blue/white screening and selected clones were suspended in 30 μl of ddH_2_O for a minimum of 1 h. Prior to sequencing, clones were screened for the expected insert size using 2 μl of bacterial water in a PCR containing M13 forward and reverse primers. For each individual, at least 20 amplified clones of the expected insert size were purified with 1 μl ExoSAP-IT (USB), and sequenced commercially (Macrogen, Korea) using M13 primers in Sanger sequencing. To validate each allelic sequence, DNA from each individual was amplified and cloned twice. Cloned sequences were retained if they occurred in at least two independent PCRs. The retained sequences were edited, assembled and aligned using Geneious v. 6 [60].

PCR products for 454 sequencing were purified using the Agencourt AMPure XP purification kit (Beckman Coulter) according to the manufacturer’s instructions. Purified products were pooled in equimolar concentrations and sequenced commercially (Macrogen, Korea) on an eighth plate on a 454 GS-FLX run.

### Bioinformatics following 454 sequencing

Following 454 sequencing, we used the jMHC software [61] to extract sequences and assign reads to individuals. Only reads showing a complete match to the forward and reverse primers were retained, ensuring that all assigned reads covered the whole amplicon. At the level of the whole dataset, we retained only sequences of exactly 159 bp which occurred in at least two independent PCRs, each represented by at least three reads [36]. Remaining sequences were assigned to individuals based on MID tags and aligned in Geneious v. 6 [60]. At the individual level, we discarded any alleles with coverage lower than 10 % of the allele with the highest coverage within each individual in order to remove less reliable sequence variants [62]. Despite the potential for excluding true alleles in individuals with very high coverage, we believe this conservative approach provides a sufficiently comprehensive assessment of the levels of diversity across species to assess patterns of MHC evolution in passerines.

### Allelic diversity and phylogenetic analyses

We calculated intraspecific nucleotide and amino acid diversity using the Kimura 2-parameter and Poisson models respectively in MEGA v.5 and estimated standard errors through 1000 bootstrap replicates [63]. We reconstructed the evolutionary relationships among MHC class II β exon 2 sequences from a) the basal passerine species in this study, and b) a wider range of avian taxa, including species from Accipitridae, Apterygidae, Ardeidae, Galliformes, Procellariformes, Spheniscidae, Strigidae, and non-Australian Passeriformes (see Additional file 6). We estimated the best-fit model of evolutionary change using jModelTest2 ([GTR + Γ; [64, 65]) based on Akaike’s Information Criterion and constructed a Bayesian phylogeny in MRBAYES v. 3.2 [66] with a Nile crocodile sequence (*Crocodylus niloticus* FJ886734) as an outgroup. Trees were sampled every 1,000 generations over 50 million generations of two simultaneous runs, with one cold and three heated Markov Coupled Monte-Carlo chains. The first 25 % of trees were discarded as burn-in and the remaining used to construct a consensus tree which was visualised in FigTree v. 1.4 [67]. We evaluated the monophyly of the passerine sequences in our dataset by comparing estimates of marginal likelihood in natural log units for positively and negatively constrained topologies [68] in MRBAYES v. 3.2. We performed topology testing, using a Bayesian stepping stone approach for 5 million generations and assessed support for the constrained topology over the negative constraint using Bayes Factors [69]. To assess phylogenetic relationships based on putatively neutral and adaptive genetic variation, we constructed Neighbour-Joining trees in MEGA v. 5 [63] using the Nei-Gojobori method with Jukes-Cantor correction based on a) non-synonymous (d_N_) and b) synonymous substitutions (d_S_). Bootstrap tests of trees were conducted using 5,000 replicates. As exon 2 of the MHC class II β genes has been demonstrated to undergo high rates of recombination and gene conversion [2, 70, 71], we complementarily utilised phylogenetic networks in addition to phylogenetic trees to visualise relationships among MHC sequences. We used the neighbour-net algorithm based on Jukes-Cantor distances in Splitstree v. 4 [45] to examine the relationships among MHC sequences from the seven basal passerine species.

## Ethics approvals

Samples were collected under permits from the Victorian Department of Environment and Primary Industries (numbers 10004294 under the *Wildlife Act* 1975 and the *National Parks Act* 1975, and NWF10455 under section 52 of the *Forest Act* 1958), the Australian Bird and Bat Banding Schemes and under approval and monitoring of Monash University ethics processes (BSCI/2007/07).

## Availability of supporting data

The sequence data supporting the results of this article are available in the Figshare digital repository and can be accessed at doi:10.4225/49/571353BCF0125.

## Additional files

Additional file 1: MHC class II β exon 2 alleles identified through cloning and 454 sequencing methods. Sample size, range and total number of alleles per species, and putative number of loci per species are given. (XLSX 8 kb) Additional file 2: Relationship between depth of coverage (total number of reads) obtained through 454 sequencing and number of alleles for each individual. (PDF 166 kb) Additional file 3: Neighbour-joining tree estimated from a) non-synonymous and b) synonymous substitutions of MHC class II β exon 2. The seven species in this study are coloured according to family and the tree rooted with *Crocodylus niloticus*. (PDF 76 kb) Additional file 4: Amino acid alignment of 159 nucleotide sites from MHC class II β exon 2. Amino acid residues distinguishing Meliphagidae, Pardalotidae and Climacteridae are highlighted in grey. (PDF 1154 kb) Additional file 5: Primer sequences trialled in this study. Standard International Union of Biochemistry (IUB) codes are used for degenerate primers. (XLSX 9 kb) Additional file 6: Avian taxa included in Bayesian phylogeny. GenBank accession numbers and family or order classification are given for species included in the Bayesian phylogeny of avian taxa (Fig. 2). (XLSX 10 kb)
