# Supplementary material for: New data from basal Australian songbird lineages show that complex structure of MHC class II β genes has early evolutionary origins within passerines
Source: BMC Evol Biol. 2016 May 21;16:112. doi: 10.1186/s12862-016-0681-5 (PMC4875725; doi:10.1186/s12862-016-0681-5)
Supplement: Additional file 2: — Relationship between depth of coverage (total number of reads) obtained through 454 sequencing and number of alleles for each individual. (PDF 166 kb) [file 12862_2016_681_MOESM2_ESM.pdf]

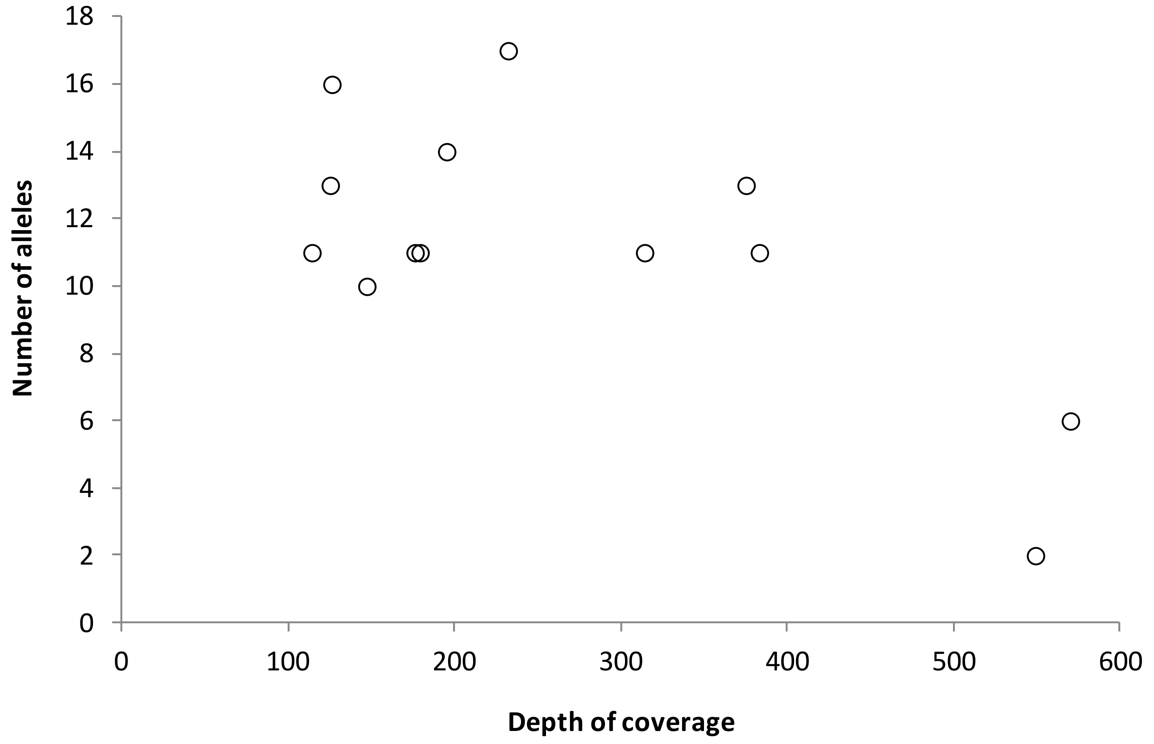

Additional file 2 Relationship between depth of coverage (total number of reads) obtained through 454 sequencing and number of alleles for each individual
