# Supplementary material for: New data from basal Australian songbird lineages show that complex structure of MHC class II β genes has early evolutionary origins within passerines
Source: BMC Evol Biol. 2016 May 21;16:112. doi: 10.1186/s12862-016-0681-5 (PMC4875725; doi:10.1186/s12862-016-0681-5)
Supplement: Additional file 3: — Neighbour-joining tree estimated from a) non-synonymous and b) synonymous substitutions of MHC class II β exon 2. The seven species in this study are coloured according to family and the tree rooted with Crocodylus niloticus. (PDF 76 kb) [file 12862_2016_681_MOESM3_ESM.pdf]

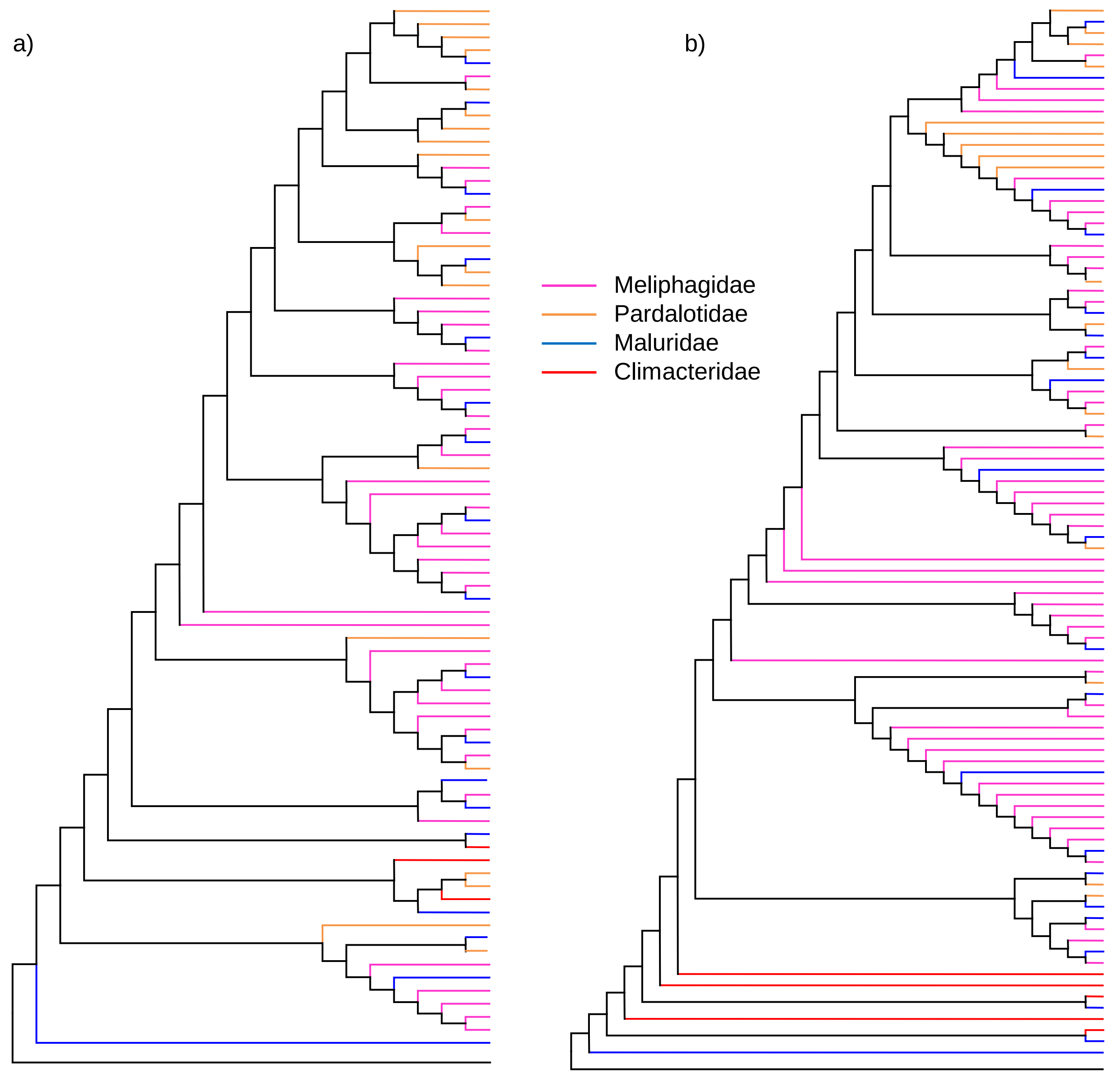

Additional file 3 Neighbour-joining tree (NJ) of MHC class II β exon 2 sequences based on a) non-synonymous and b) synonymous substitutions from the seven species in this study, rooted with *Crocodylus niloticus*
