# Supplementary material for: New data from basal Australian songbird lineages show that complex structure of MHC class II β genes has early evolutionary origins within passerines
Source: BMC Evol Biol. 2016 May 21;16:112. doi: 10.1186/s12862-016-0681-5 (PMC4875725; doi:10.1186/s12862-016-0681-5)
Supplement: Additional file 4: — Amino acid alignment of 159 nucleotide sites from MHC class II β exon 2. Amino acid residues distinguishing Meliphagidae, Pardalotidae and Climacteridae are highlighted in grey. (PDF 1154 kb) [file 12862_2016_681_MOESM4_ESM.pdf]

Clpi01	E	R	V	R	F	V	E	R	Y	I	H	N	R	E	K	Y	V	I	L	D	S	D	V	G	H	F	V	G	D	T	P	Y	G	E	F	Q	A	R	Y	W	N	S	D	P	G	I	L	E	N	A	R	T	A
Clpi02	.	.	.	.	.	.	.	.	.	.	.	.	.	.	.	.	.	.	F	.	.	.	.	.	.	.	.	.	.	.	.	.	.	.	.	.	.	.	.	.	.	.	.	.	.	.	.	.	.	.	Q	.	.
Clpi03	.	.	.	.	.	.	D	.	.	.	Y	.	.	.	Q	.	.	.	F	.	.	.	.	.	.	Y	.	.	.	.	.	R	.	.	K	V	.	.	.	.	.	.	L	.	E	W	I	.	Y	K	.	G	Q
Clpi04	.	.	.	.	.	.	.	.	H	.	Y	.	.	.	Q	.	T	.	F	.	.	.	.	.	.	Y	.	.	.	.	.	.	.	.	.	.	.	.	.	.	.	.	.	.	.	.	.	.	.	.	.	.	.
Clpi05	.	.	.	.	.	.	.	.	H	.	Y	.	.	.	Q	.	.	.	F	.	.	.	.	.	.	.	.	.	.	.	.	R	.	.	K	V	.	.	.	.	.	.	L	.	E	R	.	.	Y	S	.	H	E
Clpi06	.	.	.	K	.	.	D	.	.	.	Y	.	.	.	Q	F	.	.	F	.	.	.	.	.	.	Y	.	.	.	.	.	.	.	.	I	.	.	.	.	.	.	.	Q	.	E	L	.	.	F	K	.	G	Q
Clpi07	.	.	.	.	Y	.	.	.	.	.	Y	.	.	.	Q	F	.	.	F	.	.	.	.	.	.	Y	.	.	.	.	.	R	.	.	K	V	.	.	N	.	.	.	L	.	E	L	.	.	.	T	.	.	.
Clpi08	.	.	.	.	.	.	.	.	.	.	Y	.	.	.	Q	.	.	.	F	.	.	.	.	.	.	.	.	.	.	N	.	R	.	.	K	V	.	.	.	.	.	.	L	.	E	R	.	.	Y	N	.	H	E
Clpi09	.	.	.	.	.	.	.	.	.	M	Y	.	.	.	Q	.	.	.	F	.	.	.	.	.	.	Y	.	.	.	.	.	R	.	.	K	V	.	.	.	.	.	.	L	.	E	R	.	.	Y	N	.	H	E
Clpi10	.	.	.	.	.	.	.	.	.	M	Y	.	.	.	Q	.	.	.	F	.	.	.	.	.	.	.	.	.	.	.	.	R	.	.	K	V	.	.	.	.	.	.	L	.	E	R	.	.	Y	N	.	H	E
Clpi11	.	.	.	K	.	.	D	.	.	.	.	.	.	.	.	.	.	.	F	.	.	.	.	.	.	.	.	.	.	.	.	.	.	.	.	.	.	.	.	.	.	.	.	.	.	.	.	.	.	.	.	.	.
Clpi13	.	.	.	K	.	.	D	.	.	.	Y	.	.	.	Q	.	.	.	F	.	.	.	.	.	.	Y	.	.	.	.	.	.	.	.	K	V	.	.	.	L	.	.	Q	.	E	L	.	.	F	K	.	G	Q
Clpi14	.	.	.	.	.	L	.	.	.	.	Y	.	.	.	Q	.	A	.	F	.	.	.	.	.	.	Y	.	.	.	.	.	F	.	.	I	.	.	.	.	.	.	.	Q	.	E	.	.	.	Y	K	.	A	E
Clpi15	.	.	.	.	.	.	D	.	.	.	Y	.	.	.	Q	.	.	.	F	.	.	.	.	.	.	Y	.	.	.	.	.	R	.	.	K	V	.	.	.	.	.	.	L	.	E	W	I	.	Y	K	.	G	Q
Clpi16	.	.	.	.	.	.	D	.	.	.	Y	.	.	.	Q	L	L	H	F	.	.	.	.	.	.	.	.	.	.	.	.	.	.	.	I	.	.	.	.	.	.	.	L	.	E	W	M	.	.	T	.	.	.
Clpi22	.	.	.	.	.	.	.	.	.	.	.	.	.	.	.	.	.	.	F	.	.	.	.	.	.	.	.	.	.	.	.	.	.	.	.	.	.	.	.	.	.	.	.	.	.	.	.	.	.	.	.	.	.
Clpi23	.	.	.	.	.	.	.	.	H	.	Y	.	.	.	Q	.	.	.	F	.	.	.	.	.	.	Y	.	.	.	.	.	F	.	.	K	V	.	.	.	.	.	.	L	.	E	Y	M	.	Y	K	.	G	N
Clpi24	.	.	.	.	.	.	.	.	.	.	.	.	.	D	Q	I	L	H	F	.	.	.	.	.	.	Y	.	.	.	.	.	.	.	.	I	.	.	.	.	.	.	.	L	.	E	L	.	.	Y	K	.	.	.
Clpi26	.	.	.	K	.	.	.	.	H	.	Y	.	.	.	Q	.	.	.	F	.	.	.	.	.	.	Y	.	.	H	.	.	H	.	.	K	A	.	.	.	.	.	.	L	.	E	R	.	.	Y	N	.	H	E
Clpi27	.	.	.	.	Y	.	.	.	H	.	Y	.	.	.	Q	.	.	.	F	.	.	.	.	.	.	Y	.	.	.	.	.	F	.	.	K	V	.	.	.	.	.	.	L	.	E	Y	M	.	Y	K	.	G	N
Clpi28	.	.	.	.	.	.	.	.	.	.	Y	.	.	.	Q	.	.	.	F	.	.	.	.	.	.	.	.	.	.	.	.	R	.	.	K	V	.	.	.	.	.	.	L	.	E	R	.	.	Y	N	.	H	E
Clpi29	.	.	.	.	.	.	.	.	.	.	Y	.	.	.	Q	.	.	.	F	.	.	.	.	.	.	Y	.	.	H	.	.	H	.	.	K	A	.	.	.	.	.	.	L	.	E	R	.	.	Y	N	.	H	E
Clpi30	.	.	.	.	.	.	.	.	.	.	.	.	.	.	.	.	.	.	F	.	.	.	.	.	.	.	.	.	.	.	.	.	.	.	.	.	.	.	.	.	.	.	.	.	.	.	.	.	.	.	.	.	.
Clpi31	.	.	A	.	.	.	.	.	.	.	.	.	.	.	.	.	.	.	F	.	.	.	.	.	.	.	.	.	.	.	.	.	.	.	.	.	.	.	.	.	.	.	.	.	.	.	.	.	.	.	.	.	.
Clpi32	.	.	.	K	.	.	D	.	.	.	Y	.	.	.	Q	F	.	.	F	.	.	.	.	.	.	Y	.	.	H	.	.	.	.	.	I	.	.	.	.	.	.	.	Q	.	E	L	.	.	F	K	.	G	Q
Clpi33	.	.	.	.	.	.	.	.	H	.	Y	.	.	.	Q	.	T	.	F	.	.	.	.	.	.	Y	.	.	.	.	.	.	.	.	K	V	.	.	.	L	.	.	L	.	E	Y	M	.	.	T	.	.	.
Clpi35	.	.	.	K	.	.	D	.	.	.	Y	.	.	.	Q	F	.	.	F	.	.	.	.	.	.	Y	.	.	.	.	.	.	.	.	K	V	.	.	.	L	.	.	Q	.	E	L	.	.	F	K	.	G	Q
Clpi39	.	.	.	.	.	.	.	.	.	.	Y	.	.	.	Q	I	.	Y	F	.	.	.	.	.	.	Y	.	.	.	.	.	R	.	.	K	A	.	.	.	L	.	.	L	.	E	R	.	.	Y	.	H	.	.
Clpi46	.	.	.	.	.	.	.	.	H	.	Y	.	.	.	Q	.	.	.	F	.	.	.	.	.	.	Y	.	.	.	.	.	F	.	.	K	V	.	.	.	.	.	.	L	.	E	F	M	.	Y	K	.	G	N
Clpi47	.	.	.	.	.	.	.	.	H	.	Y	.	.	.	Q	.	A	.	F	.	.	.	.	.	.	Y	.	.	.	.	.	F	.	.	K	V	.	.	.	.	.	.	L	.	E	Y	M	.	Y	K	.	G	N
Lifu01	D	G	.	.	.	.	K	.	F	.	Y	.	.	.	Q	.	.	H	F	.	.	.	.	.	Q	.	.	.	.	.	R	F	.	.	K	V	.	.	H	.	.	.	.	.	E	W	.	.	Y	R	.	A	.
Lifu03	.	K	.	.	.	M	.	.	Q	M	Y	.	.	L	E	D	.	R	F	.	.	.	.	.	K	.	.	.	F	.	.	.	.	.	K	.	.	K	.	.	.	.	.	Q	A	W	M	.	.	R	.	A	Q
Lifu04	.	K	.	.	L	L	D	.	.	.	Y	.	.	L	E	D	.	R	F	.	.	.	.	.	K	.	.	.	L	.	.	F	.	K	K	N	.	E	R	L	.	.	.	.	N	R	.	.	Y	K	.	A	.
Lifu05	.	K	.	.	.	M	.	.	.	.	Y	.	.	V	E	F	L	R	F	.	.	.	.	.	K	Y	E	.	F	.	.	L	.	.	K	N	.	E	R	A	.	.	.	.	N	R	.	.	H	.	Q	.	.
Lifu06	.	K	.	.	.	L	Q	.	.	.	Y	.	.	L	E	G	M	R	F	.	.	.	.	.	K	H	.	.	F	.	.	F	.	K	K	N	.	E	R	.	.	.	.	.	N	.	.	.	H	R	Q	A	E

Lifu07	.	K	.	.	L	L	D	.	.	.	Y	.	.	V	E	.	A	R	F	.	.	.	.	.	K	Y	.	.	F	.	.	F	.	.	K	N	.	E	R	.	.	.	.	.	N	.	.	.	H	R	Q	A	.
Lifu08	.	K	.	.	.	L	D	.	.	.	Y	.	.	V	E	L	M	R	F	.	.	.	.	.	K	Y	.	.	F	.	.	L	.	.	E	N	.	E	R	L	.	.	.	.	N	R	.	.	Y	.	Q	.	.
Lifu09	.	K	.	.	L	L	D	.	.	.	Y	.	.	V	E	L	T	R	F	.	.	.	.	.	K	Y	.	.	F	.	.	.	.	.	K	.	.	K	.	.	.	.	N	.	N	R	.	.	H	.	Q	.	.
Lifu10	.	K	.	.	.	M	.	.	H	.	Y	.	.	V	E	D	L	R	F	.	.	.	.	.	K	.	.	.	F	.	.	F	.	.	K	I	.	E	R	L	.	.	.	.	N	R	.	.	Y	.	Q	.	.
Lifu11	D	G	.	.	.	.	K	.	F	.	Y	.	.	.	Q	.	.	H	F	.	.	.	.	.	Q	.	.	.	.	.	R	F	.	.	K	V	.	.	H	.	.	.	.	.	E	W	.	.	Y	R	.	A	.
Lifu12	.	K	.	.	.	M	.	.	H	.	Y	.	.	V	E	.	M	R	F	.	.	.	.	.	K	.	.	.	F	.	.	F	.	.	K	I	.	E	R	V	.	.	.	.	N	.	.	.	H	T	Q	S	.
Lifu13	.	K	.	.	.	M	.	.	Q	M	Y	.	.	V	E	.	A	R	F	.	.	.	.	.	K	Y	.	.	F	.	.	.	.	.	K	W	.	K	.	.	.	.	.	Q	A	W	M	.	.	R	.	A	Q
Lifu14	.	K	.	.	.	.	D	.	.	.	Y	.	.	V	E	L	T	R	F	.	.	.	.	.	K	Y	.	.	F	.	.	.	.	.	K	.	.	K	.	.	.	.	N	.	N	R	.	.	H	.	Q	.	.
Lifu15	.	K	.	.	.	M	A	.	T	.	Y	.	.	V	E	D	R	R	F	.	.	.	.	.	K	Y	.	.	F	.	.	.	.	.	K	I	.	E	R	L	.	.	.	.	N	R	.	.	Y	.	Q	.	.
Lifu16	.	K	.	.	.	M	A	.	T	.	Y	.	.	V	E	D	R	R	F	.	.	.	.	.	K	Y	.	.	F	.	.	.	.	.	K	I	.	E	R	A	.	.	.	.	N	R	.	.	Y	.	Q	.	.
Lifu17	.	K	.	.	.	L	D	.	.	.	Y	.	.	V	E	D	.	R	F	.	.	.	.	.	K	Y	.	.	F	.	.	F	.	K	K	.	.	K	.	R	.	.	.	.	N	L	.	D	Y	K	.	A	L
Lifu18	D	G	.	.	.	.	K	.	F	.	Y	.	.	.	Q	.	.	H	F	.	.	.	.	.	Q	.	.	.	.	.	R	F	.	.	K	V	.	.	R	L	P	G	T	G	T	A	T	R	.	G	W	S	T
Lifu20	.	K	.	.	.	.	V	.	A	.	Y	.	G	V	E	G	.	R	F	N	.	N	.	.	K	Y	.	.	F	.	.	.	.	.	K	W	.	Q	H	.	.	.	N	A	N	E	M	.	Y	K	T	G	S
Lifu21	.	K	.	.	.	.	V	.	A	.	Y	.	G	V	E	G	.	R	F	N	.	N	.	.	K	Y	.	.	F	.	.	.	.	.	K	W	.	Q	H	.	.	.	N	A	N	E	M	.	Y	K	M	G	S
Lifu22	.	K	.	.	.	L	D	.	.	.	Y	.	.	V	E	.	A	R	F	.	.	.	.	.	K	Y	.	.	F	.	.	.	.	.	K	.	.	E	.	.	.	G	N	.	D	.	M	.	Y	K	.	A	Q
Lifu23	.	K	.	.	.	L	Q	.	.	.	Y	.	.	L	E	D	.	R	F	.	.	.	.	.	K	.	.	.	F	.	.	A	.	D	K	W	.	Q	D	.	.	.	.	E	A	R	M	.	Y	T	.	A	Q
Lifu26	.	K	.	.	L	L	A	.	.	.	Y	.	.	V	E	.	A	R	F	.	.	.	.	.	K	Y	.	.	F	.	.	.	.	.	K	N	.	E	.	.	.	.	.	.	N	R	.	.	Y	.	Q	.	.
Lifu29	.	K	.	.	Y	.	D	.	.	.	Y	.	.	V	E	L	T	R	F	.	.	.	.	.	K	Y	.	.	F	.	.	.	.	.	K	N	.	E	.	.	.	.	N	.	N	R	.	.	R	.	Q	A	E
Lifu30	.	K	.	.	.	L	D	.	H	.	Y	.	.	V	E	L	M	R	F	.	.	.	.	.	K	Y	.	.	F	.	.	F	.	.	K	I	.	E	R	A	.	.	.	.	N	R	.	.	Y	.	Q	.	.
Lime01	.	K	.	.	Y	M	.	.	Q	M	Y	.	.	V	E	.	L	R	F	.	.	.	.	.	K	.	.	.	F	.	.	.	.	.	K	.	.	K	.	.	.	.	.	Q	A	W	M	.	Y	K	.	.	.
Lime02	D	G	.	.	.	.	K	.	F	.	Y	.	.	.	Q	.	.	H	F	.	.	.	.	.	Q	.	.	.	.	.	.	F	.	.	K	V	.	.	H	.	.	.	.	.	E	W	.	.	Y	R	.	A	.
Lime03	.	K	.	.	.	M	.	.	F	.	Y	.	.	V	E	.	M	R	F	.	.	.	E	R	K	Y	.	.	F	.	.	.	.	.	K	.	.	N	.	A	.	.	.	Q	A	W	M	.	Y	K	.	.	.
Lime04	.	K	.	.	.	L	Q	.	.	.	Y	.	.	V	E	.	L	R	.	.	.	.	E	.	K	Y	L	.	F	.	.	F	.	.	K	W	V	K	.	.	.	T	N	.	N	L	.	.	Y	V	Q	.	E
Lime05	.	K	.	.	.	L	Q	.	.	.	Y	.	.	V	E	.	T	R	F	.	.	.	.	.	K	.	.	.	F	.	.	D	.	.	K	W	.	K	.	.	.	.	.	K	A	G	M	.	Y	T	.	A	Q
Lime06	.	K	.	.	H	.	.	.	.	.	Y	.	.	V	E	F	.	R	F	.	.	.	.	.	K	Y	.	.	F	.	.	D	.	.	K	N	.	E	R	.	.	.	.	.	D	.	.	.	H	V	.	S	L
Lime07	.	K	.	.	.	L	Q	.	.	.	Y	.	.	V	E	.	T	R	F	.	.	.	.	R	K	.	.	.	F	.	.	D	.	D	K	W	.	K	.	.	.	.	.	K	A	G	M	.	Y	T	.	A	Q
Lime08	.	K	.	.	L	L	D	.	F	.	Y	.	.	L	E	D	.	R	F	.	.	.	.	.	K	Y	.	.	F	.	.	.	.	.	K	L	.	K	.	.	.	.	.	.	A	R	M	.	Y	.	Q	A	G
Lime09	.	K	.	.	.	L	D	.	H	.	Y	.	.	V	E	.	.	R	F	.	.	.	.	.	K	Y	.	.	F	.	.	F	.	.	K	.	.	K	R	L	.	.	.	E	A	R	M	.	Y	T	.	A	Q
Lime10	.	K	.	.	L	L	D	.	.	.	Y	.	.	L	E	D	.	R	F	.	.	.	.	.	K	Y	.	.	F	.	.	A	.	.	K	W	.	Q	D	.	.	.	.	E	A	G	M	.	H	T	.	.	.
Lime11	.	K	.	.	Y	M	.	.	H	M	Y	.	.	L	E	H	L	R	F	.	.	.	.	.	K	.	.	.	F	.	.	.	.	.	K	N	.	E	R	L	.	.	.	.	N	.	.	.	Y	D	Q	A	E
Lime12	.	K	.	.	.	.	G	S	A	F	Y	.	.	V	E	F	M	R	F	.	.	.	.	.	K	Y	.	.	F	.	.	F	.	.	K	N	.	E	R	.	.	.	.	.	D	.	.	.	H	.	.	S	L
Lime13	.	K	.	.	Y	M	D	.	N	.	Y	.	.	V	E	L	.	S	F	.	.	N	.	.	K	Y	.	.	F	.	.	F	.	.	K	.	.	E	.	.	.	.	.	.	D	R	.	.	Y	Q	.	.	S
Lime14	.	K	.	.	.	L	Q	.	.	.	Y	.	.	V	E	D	.	R	F	.	.	.	.	.	K	.	.	.	F	.	.	F	.	.	K	.	.	K	R	A	.	.	.	E	A	R	M	.	Y	T	Q	A	.
Lime15	.	K	.	.	.	L	.	.	.	.	Y	.	.	V	E	.	A	R	F	.	.	.	.	.	K	Y	.	.	F	.	.	.	.	.	K	.	.	K	.	.	.	.	N	.	D	.	M	.	Y	R	.	A	.
Lime16	.	K	.	.	L	L	D	.	.	.	Y	.	.	V	E	.	A	R	F	.	.	.	.	.	K	.	.	.	F	.	.	.	.	.	K	.	.	K	C	.	.	.	N	.	D	.	M	.	Y	R	.	A	.

Lime18	. K . . . L . . Q M Y . . V E L . R F . . . . . K . . . F . . F . . K . . K . . . . . Q A W M . . R . A Q
Lime19	. K . . L . V . T M Y . . V E L K R F . . . . . K Y E . F . . . . . K W . Q D L . R . S N L . . Y T . . .
Lime21	. K . . L L Q . . . Y . . V E . A R F . . . . . K Y . . F . L . . . K . . K . . . . . E A G M . Y T Q A .
Lime22	. K . . . L D . . . Y . . V E . A R F . . N . . K Y . . F . . A . . K . . E . . . . . E A G M . Y T . . .
Lime23	. K . . L L Q . . . Y . . V E . A R F . . . . . K Y . . F . L . . . K . . K C . . . . E A G M . Y T Q A .
Lime28	. K . . . M V . . . Y . . V E . A R F . . . . . K . . . F . . . . . K N . E R . . . E . N . . . Y V Q . .
Lime29	. K . . . M . . F . Y . . V E . M R F . . . E R K Y . . F . . . . . K . . N . A . . . Q A W M . Y K . . .
Lime30	. K . . Y M Q K L . Y . . V E . A R F . N N . . K Y . . F . . . . . K . . K . . . N L . V F M . G R . . .
Lime31	. K . . . L Q . . . Y . . V E . L R . . . . E . K Y L . F . . F . . K W V K . . . T N . N L . . Y V Q . E
Lime32	. K . . Y M . . Q M Y . . L E H L R F . . . . . K Y . . F . . L . . E N . E R L . . . E A R M . Y I . A Q
Lime33	. K . . . . V . A . Y . C V E G . R F N . N . . K Y . . F . . . . . K W . Q H . . . N A N E M . Y K T G S
Lime34	. K . . . L . . . . Y . . V E . L R F . . . . . K Y . . F . . . . . K . . K . . . . . E A G M . Y T Q . E
Lime36	. K . . . M . . . . Y . . V E . L R . . . . E . K Y L . F . . F . . K W V K . . . T N . N L . . Y V Q . E
Lime37	. K . . Y . D . S . Y . . V E F L R F . . Y . . K H . . F . S F . . K I . Q C L . . . S D L M . R K Q A .
Lime38	. K . M . . V . A V Y S G V E G . R F . . N . . K Y . . F . . . . . K W . Q H . . . N T N E M . Y K M G S
Lime39	. K . . . . V . A . Y . G V E G . R F . . N . . K Y . . F . . . . . K W . Q H . . . N A N E M . Y K T G S
Lime40	. K . M . . V . A . Y S G V E G . R F . . N . . K Y . . F . . . . . K W . Q H . . . N A N E M . Y K M G S
Lime42	. K . M . . V . A V Y S G V E G . R F . . N . . K Y . . F . . . . . K W . Q H . . . N A N E M . Y K M G S
Lime44	. K . . . M V . F . Y . . V E F A R F . . . . . K Y . . F . . F . . K . . K . . . . . . D . M . Y R . A Q
Lime45	. K . . . L Q . . . Y . . V E . L R . . . . E . K Y L . F . . F . . K W V K . . . T N . N L . . Y V Q . E
Lime48	. K . . . L D . S . Y . . V E . . R F . . . . . K Y . . F . . . . . K V . E . . . . . E A W M . . T . A Q
Lipe01	D G . . . . K . F . Y . . . Q . . H F . . . . . Q . . . . . . F . . K I . . H . . . . . E W . . Y R . A .
Lipe02	. K . . Y M . . Q M Y . . V E . M R F . . . . . K H E . F . . . . . K V . K R L . . . . S W M . Y . . . T
Lipe03	. K . . Y M . . . . Y . . G E . A R F . . . . . K Y . . F . . L . . K . . K . . . . . E A G M . Y T . . .
Lipe04	. K . . . M . . H . Y . . V E L M R F . . . . . K . . . F . . F . . K I . E R A . . . . N R . . Y . Q . .
Lipe05	. K . . L . D . . . Y . . L E D . R F . . . . . K H . . F . . H . . K . . K C . . . . E A W M . . T . A .
Lipe06	. K . . Y . H . . . Y . . V E L T M F . . . . . K Y . . F . . L . . K W . K . . . . N . D F I . I K . A E
Lipe07	. K . . Y L D . . . Y . . V E L A R F . . . . . K Y . . F . . . . . K . . K . . . G N . D . M . . R Q A E
Lipe08	. K . K Y . A . V . Y . . V E . L R F . . . . . K Y . . F . . . . D R N . E R L . . N . N E M . Y R Q A E
Lipe09	. K . . Y M V . L . Y . . V E . A R F . . . . . K Y . . F . . . . . K . . K . R . . N . S W M . Y K . A .
Lipe10	. K . . Y . Q . . . Y . . V E . . R F . . . . . K Y . . F . L . . . K N . K R . . . . . D E . . Y R . A E
Lipe11	. K . . Y . N . . . Y . . V E . T R F . . . . . K . . . F . . . . . K . . K . R . . L . D F M . R I . . S
Lipe13	. K . . . . Y . . V E . . R F . . . . . K . . . F . . . . . K . . E . A . . . . A W M . Y K . A E
Lipe16	. K . . . . V . A . Y . G V E G . R F N . N . . K Y . . F . . . . . K W . Q H . . . N A N E M . Y K T G S
Lipe18	. K . . . . V . A . Y . C V E G . R F N . N . . K Y . . F . . . . . K W . Q H . . . N A N E M . Y K T G S

Lipe19	.	K	.	.	.	L	D	.	S	.	Y	.	.	V	E	.	.	R	F	.	.	.	.	.	K	Y	.	.	F	.	.	.	.	.	K	V	.	E	.	.	.	.	.	E	A	W	M	.	.	T	.	A	Q
Lipe21	.	K	.	.	.	L	Q	.	.	.	Y	.	.	L	E	D	.	R	F	.	.	.	.	.	K	.	.	.	F	.	.	A	.	D	K	W	.	Q	D	.	.	.	.	E	A	R	M	.	Y	T	.	A	Q
Lipe22	.	K	.	.	.	L	D	.	.	.	Y	.	.	V	E	.	A	R	F	.	.	.	.	.	K	Y	.	.	F	.	.	.	.	.	K	.	.	E	.	.	.	G	N	.	D	.	M	.	Y	K	.	A	Q
Lipe24	.	K	.	.	.	.	V	.	A	.	Y	.	G	V	E	G	.	R	F	N	.	N	.	.	K	Y	.	.	F	.	.	.	.	.	K	W	.	Q	H	.	.	.	N	A	N	E	M	.	Y	K	T	G	S
Lipe25	.	K	.	.	.	.	V	.	A	.	Y	.	G	V	E	G	.	R	F	N	.	N	.	.	K	Y	.	.	F	.	.	.	.	.	K	W	.	Q	H	.	.	.	N	A	N	E	M	.	Y	K	T	G	S
Macy01	.	K	.	.	Y	F	.	.	H	.	Y	.	.	.	Q	L	M	H	F	.	.	.	.	.	L	Y	.	.	.	.	.	.	.	.	K	.	.	.	.	Y	.	.	Q	A	E	Y	.	.	Y	T	.	G	V
Macy02	.	K	.	.	Y	M	.	.	H	F	Y	.	.	V	E	F	L	R	F	.	.	.	.	.	D	.	.	.	F	.	.	W	.	.	K	N	.	.	.	.	.	.	Q	.	E	W	M	.	.	R	.	.	Q
Macy03	.	N	.	.	L	M	V	.	A	.	Y	.	.	.	Q	.	I	H	F	.	.	.	.	.	D	.	.	.	.	.	.	.	.	.	K	V	.	.	.	.	.	.	Q	A	V	Y	M	.	S	M	.	.	.
Macy04	.	K	M	.	L	.	A	.	A	M	Y	.	.	.	Q	L	L	H	F	.	.	.	.	.	E	.	.	.	.	.	.	F	.	.	K	.	.	.	.	.	.	.	Q	.	E	Y	M	.	G	K	.	A	E
Macy05	.	K	.	.	Y	M	.	.	Q	F	Y	.	.	V	E	F	L	R	F	.	.	.	.	.	D	.	.	.	F	.	.	W	.	.	K	N	.	.	.	.	.	.	Q	.	E	W	M	.	.	R	.	.	Q
Macy06	.	K	M	.	L	L	A	.	A	M	Y	.	.	.	Q	L	L	H	F	.	.	.	.	.	L	.	.	.	.	.	.	W	.	.	K	.	.	.	.	Y	.	.	K	.	E	.	.	.	Y	N	.	G	I
Macy07	.	K	.	.	Y	.	H	.	H	L	Y	.	.	V	E	F	L	R	F	.	.	.	.	.	E	Y	.	.	F	.	.	W	.	.	K	.	.	P	.	.	.	.	L	.	E	W	M	.	R	K	.	A	E
Macy08	A	.	.	.	.	.	K	.	F	.	Y	.	.	.	Q	.	.	H	F	.	.	.	.	.	L	.	.	.	H	.	.	.	.	.	K	I	V	G	.	.	.	.	Q	L	E	W	M	.	Y	R	.	A	.
Macy09	.	K	.	.	Y	.	Q	.	.	.	Y	.	.	V	E	F	A	R	F	E	.	.	L	.	D	.	.	.	F	.	.	W	.	.	K	N	.	.	H	.	.	.	L	.	E	R	.	A	H	K	.	G	Q
Macy10	K	K	.	.	.	.	V	.	H	.	Y	.	.	V	E	F	.	R	F	.	.	.	.	.	Q	.	.	.	F	.	.	.	.	.	K	.	.	.	.	.	.	.	Q	A	E	Y	.	.	Y	T	.	G	V
Macy11	K	K	.	.	.	M	V	.	H	.	Y	.	.	V	E	F	.	R	F	.	.	.	.	.	Q	.	.	.	F	.	.	.	.	.	K	.	.	.	.	.	.	.	Q	A	E	Y	.	.	Y	T	.	G	V
Macy12	A	.	.	.	.	.	K	.	F	.	Y	.	.	.	Q	.	.	H	F	.	.	.	.	.	L	.	.	.	H	.	.	.	.	.	K	I	V	.	F	.	.	.	Q	L	E	W	M	.	Y	R	.	A	.
Macy13	A	.	.	.	.	.	K	.	F	.	Y	.	.	.	Q	.	.	H	F	.	.	.	.	.	L	.	.	.	H	.	.	.	.	.	K	I	V	.	F	.	.	.	Q	L	E	W	M	.	Y	R	.	A	.
Macy14	.	K	.	.	Y	.	Q	.	L	.	Y	.	.	V	E	F	A	R	F	.	.	.	.	.	E	.	.	.	F	.	.	.	.	.	K	.	.	.	.	R	.	G	L	A	D	F	M	.	T	Q	.	.	S
Macy15	.	K	.	.	Y	.	Q	.	.	.	Y	.	.	V	E	F	A	R	F	E	.	.	L	.	D	.	.	.	F	.	.	W	.	.	K	N	.	.	.	.	.	.	L	.	E	R	.	A	H	K	.	G	Q
Macy16	.	K	.	.	Y	.	Q	.	.	.	Y	.	.	.	E	F	A	R	F	E	.	.	L	.	D	.	.	.	F	.	.	W	.	.	K	N	.	.	H	.	.	.	L	.	E	R	.	A	H	K	.	G	Q
Macy17	.	K	.	.	.	L	Q	.	.	.	Y	.	.	L	E	D	.	R	F	.	.	.	.	.	K	.	.	.	F	.	.	A	.	D	K	W	.	Q	D	.	.	.	.	E	A	R	M	.	Y	T	.	A	Q
Macy18	.	K	.	.	.	.	Q	.	F	.	Y	.	.	V	E	.	A	R	F	.	.	.	.	.	E	.	.	.	F	.	.	H	.	.	K	N	.	.	.	.	.	G	Q	S	D	L	M	.	R	P	.	.	.
Macy19	.	.	.	.	Y	.	Q	S	R	.	Y	.	.	.	L	.	.	T	F	.	.	.	.	.	.	.	.	.	F	.	G	N	.	.	I	.	.	.	.	.	.	.	N	.	V	.	.	.	Q	I	Q	.	.
Macy20	.	K	.	.	.	L	Q	.	.	.	Y	.	.	V	E	.	L	R	.	.	.	.	E	.	K	Y	L	.	F	.	.	F	.	.	K	W	V	K	.	.	.	T	N	.	N	L	.	.	Y	V	Q	.	E
Macy21	.	K	.	.	L	L	D	.	.	.	Y	.	.	L	E	D	.	R	F	.	.	.	.	.	K	Y	.	.	F	.	.	A	.	.	K	W	.	Q	D	.	.	.	.	E	A	G	M	.	H	T	.	.	.
Macy22	.	K	.	.	Y	.	D	.	S	.	Y	.	.	V	E	F	L	R	F	.	.	Y	.	.	K	H	.	.	F	.	S	F	.	.	K	I	.	Q	C	L	.	.	.	S	D	L	M	.	R	K	Q	A	.
Macy23	.	K	.	.	.	L	.	.	.	.	Y	.	.	V	E	.	L	R	F	.	.	.	.	.	K	Y	.	.	F	.	.	.	.	.	K	.	.	K	.	.	.	.	.	E	A	G	M	.	Y	T	Q	.	E
Macy24	.	.	.	.	Y	.	Q	S	R	.	Y	.	.	.	L	.	L	T	F	.	.	.	.	.	.	.	.	.	F	.	G	N	.	.	I	.	.	.	.	.	.	.	.	.	V	.	.	.	Q	I	Q	.	.
Macy25	.	K	.	M	.	.	V	.	A	V	Y	S	G	V	E	G	.	R	F	.	.	N	.	.	K	Y	.	.	F	.	.	.	.	.	K	W	.	Q	H	.	.	.	N	A	N	E	M	.	Y	K	M	G	S
Macy26	D	G	.	.	.	.	K	.	F	.	Y	.	.	.	Q	.	.	H	F	.	.	.	.	.	Q	.	.	.	.	.	.	F	.	.	K	V	.	.	H	.	.	.	.	.	E	W	.	.	Y	R	.	A	.
Macy27	.	K	.	.	.	.	V	.	A	.	Y	.	G	V	E	G	.	R	F	.	.	N	.	.	K	Y	.	.	F	.	.	.	.	.	K	W	.	Q	H	.	.	.	N	A	N	E	M	.	Y	K	T	G	S
Macy29	.	K	.	.	Y	L	D	.	.	.	Y	.	.	V	E	L	A	R	F	.	.	.	.	.	K	Y	.	.	F	.	.	.	.	.	K	.	.	K	.	.	.	G	N	.	D	.	M	.	.	R	Q	A	E
Macy33	.	K	.	.	A	M	D	.	L	F	Y	.	.	V	E	.	M	R	F	.	.	.	.	.	E	Y	.	.	F	.	.	W	.	.	R	A	.	.	N	.	K	K	L	.	E	F	M	.	R	.	.	.	.
Macy36	.	K	.	.	Y	.	Q	.	.	.	Y	.	.	V	E	F	A	R	F	E	.	.	L	.	D	.	.	.	F	.	.	W	.	.	K	N	.	.	H	.	.	.	L	.	E	R	.	A	H	K	.	G	Q

Macy37	.	K	.	.	.	L	Q	.	.	.	Y	.	.	V	E	.	L	R	.	.	.	.	E	.	K	Y	L	.	F	.	.	F	.	.	K	W	V	K	.	.	.	T	N	.	N	L	.	.	Y	V	Q	.	E
Papu01	.	K	.	.	.	.	D	.	.	.	Y	.	.	V	E	.	A	R	F	.	.	.	.	.	E	.	.	.	F	.	.	N	.	.	K	.	.	.	.	.	.	G	Q	.	D	V	M	.	T	R	.	.	.
Papu02	.	K	.	.	L	.	D	.	.	.	Y	.	.	V	E	.	A	R	F	.	.	.	.	.	E	.	.	.	F	.	.	F	.	.	K	.	.	.	.	K	.	G	L	A	D	Y	M	.	T	L	.	.	.
Papu03	.	K	.	.	Y	.	Q	.	S	.	Y	.	.	V	E	D	.	R	F	.	.	.	.	.	E	.	.	.	F	.	.	F	.	.	K	.	.	.	.	R	.	G	L	A	D	Y	M	.	T	R	.	.	S
Papu04	.	K	.	.	.	.	L	.	N	.	Y	.	.	V	E	D	.	R	F	.	.	.	.	.	E	Y	.	.	F	.	.	D	.	.	K	.	.	.	.	.	.	G	L	A	D	L	M	.	T	R	.	.	.
Papu05	.	K	.	.	Y	.	D	.	F	.	Y	.	.	V	E	.	.	R	F	.	.	.	.	.	E	Y	.	.	F	.	.	.	.	.	K	.	.	.	.	R	.	G	L	A	D	Y	M	.	T	K	.	.	.
Papu06	.	K	.	.	.	.	L	.	.	.	Y	.	.	V	E	D	.	R	F	.	.	.	.	.	E	Y	.	.	F	.	.	.	.	.	K	.	.	.	H	.	.	G	L	A	D	L	M	.	R	R	.	.	E
Papu07	.	K	.	.	.	.	L	.	.	.	Y	.	.	V	E	D	.	R	F	.	.	.	.	.	E	Y	.	.	F	.	.	D	.	.	K	.	.	.	H	.	.	G	L	A	D	L	M	.	R	R	.	.	Q
Papu08	.	K	.	.	.	.	V	.	N	.	Y	.	.	V	E	.	A	R	F	.	.	.	.	.	E	.	.	.	F	.	.	F	.	.	K	.	.	.	.	K	.	G	Q	A	D	Y	M	.	T	L	.	.	.
Papu09	.	K	.	.	.	.	D	.	.	.	Y	.	.	V	E	.	.	R	F	.	.	.	.	.	E	.	.	.	F	.	.	F	.	.	K	.	.	.	.	K	.	G	L	A	D	F	M	K	Y	T	.	.	.
Papu10	.	K	.	.	.	.	Q	.	L	.	Y	.	.	V	E	.	.	R	F	.	.	.	.	.	E	.	.	.	F	.	.	F	V	.	K	.	.	.	.	K	.	G	L	A	D	F	M	K	Y	T	.	.	.
Papu11	.	K	.	.	Y	.	Q	.	N	.	Y	.	.	V	D	I	L	R	F	.	.	.	.	.	E	Y	.	.	F	.	.	F	.	.	K	.	.	.	.	.	.	G	L	A	D	F	M	.	R	R	.	.	.
Papu12	.	K	.	.	Y	.	D	.	F	.	Y	.	.	V	E	.	A	R	F	.	.	.	.	.	E	Y	.	.	F	.	.	.	.	.	K	.	.	.	.	R	.	G	L	A	D	Y	M	.	T	Q	.	.	.
Papu13	.	K	.	.	.	.	D	.	.	.	Y	.	.	V	E	.	A	R	Y	.	.	.	.	.	E	.	.	.	F	.	.	N	.	.	K	.	.	.	.	.	.	G	Q	.	D	V	M	.	T	R	.	.	.
Papu14	.	K	.	.	.	.	L	.	.	.	Y	.	.	V	E	D	.	R	F	.	.	.	.	.	E	Y	.	.	F	.	.	D	.	.	K	.	.	.	.	.	.	G	L	A	D	L	M	.	R	R	.	.	.
Papu16	.	K	.	.	.	.	L	.	.	.	Y	.	.	V	E	.	A	R	F	.	.	.	.	.	E	Y	.	.	F	.	.	.	.	.	K	.	.	W	H	.	.	G	L	A	D	L	M	.	R	R	.	.	E
Papu17	.	K	.	.	Y	.	Q	.	N	.	Y	.	.	V	D	I	L	R	F	.	.	.	.	.	E	Y	.	.	F	.	.	F	.	.	K	.	.	.	.	.	.	G	L	A	D	Y	M	.	I	R	.	.	.
Papu18	.	K	.	.	L	.	.	.	R	.	Y	.	.	V	E	.	A	R	F	.	.	.	.	.	E	Y	.	.	F	.	.	.	.	.	E	.	.	.	.	.	.	G	L	A	D	L	M	.	T	R	.	.	.
Papu19	.	K	.	.	.	.	D	.	F	.	Y	.	.	V	E	.	.	R	F	.	.	.	.	.	E	Y	.	.	F	.	.	F	.	.	K	.	.	.	.	K	.	G	L	A	D	F	M	.	Y	T	.	.	.
Papu20	.	K	.	.	.	.	D	.	F	.	Y	.	.	V	E	.	A	R	F	.	.	.	.	.	E	Y	.	.	F	.	.	F	.	.	K	.	.	.	.	K	.	G	L	A	D	F	M	.	Y	T	.	.	.
Papu22	.	K	.	.	Y	.	.	.	.	.	Y	.	.	V	E	.	E	R	F	.	.	Y	.	.	E	.	.	.	F	.	.	D	.	.	K	.	.	.	.	T	.	G	L	A	D	Y	M	.	H	T	.	.	.
Papu25	.	K	.	.	Y	.	D	.	.	.	Y	.	.	V	E	.	A	R	F	.	.	.	.	.	E	.	.	.	F	.	.	F	.	.	K	.	.	.	.	K	K	G	L	A	D	Y	M	.	T	R	.	.	.
Papu26	.	K	.	.	Y	.	.	.	.	.	Y	.	.	V	E	.	A	R	F	.	.	.	.	.	E	.	.	.	F	.	.	D	.	.	K	H	.	.	.	T	.	G	L	A	D	Y	M	.	H	T	.	.	.
Papu28	.	K	.	.	L	L	V	K	.	.	Y	.	.	.	Q	L	.	H	F	.	.	.	.	.	.	.	.	.	.	.	.	F	.	.	K	V	.	.	N	.	D	.	N	.	V	.	M	.	R	R	.	A	E
Past01	.	K	.	.	Y	.	D	.	.	.	Y	.	.	V	E	.	A	R	F	.	.	.	.	.	E	.	.	.	F	.	.	N	.	.	K	.	.	.	.	.	.	G	L	A	D	F	M	.	R	L	W	.	S
Past02	.	K	.	.	Y	.	.	K	R	.	Y	.	.	V	E	.	A	R	F	.	.	.	.	.	E	.	.	.	F	.	.	H	.	.	K	N	.	.	.	.	.	.	Q	.	D	Y	M	.	S	Q	.	.	T
Past03	.	K	.	.	Y	.	D	.	.	.	Y	.	.	V	E	.	A	R	F	.	.	.	.	.	E	.	.	.	F	.	.	.	.	.	K	.	.	.	.	K	.	G	Q	.	A	Y	M	.	R	L	.	.	.
Past04	.	K	.	.	Y	.	D	K	.	.	Y	.	.	V	E	.	A	R	F	.	.	.	.	.	E	.	.	.	F	.	.	.	.	.	K	.	.	.	.	R	.	G	L	A	D	Y	M	.	T	R	.	.	S
Past05	.	K	.	.	Y	.	D	.	.	.	Y	.	.	V	E	.	L	T	F	.	.	.	.	.	E	.	.	.	F	.	.	D	.	.	K	N	.	.	.	F	.	G	L	G	H	Y	M	.	Q	L	.	.	.
Past06	.	K	.	.	.	.	V	.	L	.	Y	.	.	V	E	.	L	R	F	.	.	.	.	.	E	Y	.	.	F	.	.	H	.	.	K	N	.	.	I	.	.	G	L	A	D	Y	M	.	T	K	.	.	.
Past07	.	K	.	.	.	.	V	.	L	.	Y	.	.	V	E	.	L	R	F	.	.	.	.	.	E	Y	.	.	F	.	.	H	.	.	K	N	.	.	I	.	.	G	L	A	D	Y	M	.	.	T	.	.	.
Past08	.	K	.	.	.	.	V	.	L	.	Y	.	.	V	E	.	A	R	F	.	.	.	.	.	E	.	.	.	F	.	.	H	.	.	K	.	.	.	.	R	.	G	L	A	D	F	M	.	.	T	.	.	.
Past09	.	K	.	.	Y	.	V	.	L	.	.	.	.	V	E	.	A	R	F	.	.	.	.	.	E	Y	.	.	F	.	.	F	.	.	K	.	.	.	.	R	.	G	L	A	D	F	M	.	T	Q	.	.	.
Past10	.	K	.	.	.	L	.	.	H	.	Y	.	.	V	E	I	L	R	F	.	.	.	.	.	E	Y	.	.	F	.	.	.	.	.	K	N	.	.	.	.	.	G	L	A	D	Y	M	.	R	K	.	.	T
Past12	.	K	.	.	.	.	V	.	Q	.	Y	.	.	V	E	.	A	R	F	.	.	.	.	.	E	.	.	.	F	.	.	F	.	.	K	.	.	.	.	R	.	G	L	A	D	F	M	.	.	T	.	.	.

Past13	.	K	.	.	Y	.	D	.	.	.	Y	.	.	V	E	.	A	R	F	.	.	.	.	.	E	.	.	.	F	.	.	.	.	.	K	.	.	.	.	R	.	G	L	A	D	Y	M	.	T	R	.	.	V
Past14	.	K	.	.	L	.	.	.	.	.	Y	.	.	V	E	.	T	R	F	.	.	.	.	.	E	.	.	.	F	.	.	F	.	.	K	.	.	.	.	R	.	G	L	A	D	F	M	.	T	R	.	.	.
Past15	.	K	.	.	.	.	D	.	.	.	Y	.	.	V	E	.	A	R	F	.	.	.	.	.	E	.	.	.	F	.	.	N	.	V	K	.	.	.	.	R	.	G	L	A	D	Y	M	.	K	R	.	.	.
Past16	.	K	.	.	.	.	D	.	.	.	Y	.	.	V	E	.	T	R	F	.	.	.	.	.	E	Y	.	.	F	.	.	.	.	.	K	.	.	.	.	R	.	G	L	A	A	F	M	.	T	Q	.	.	.
Past17	.	K	.	.	.	.	V	.	Q	.	Y	.	.	V	E	.	A	R	F	.	.	.	.	.	E	.	.	.	F	.	.	F	.	.	K	.	.	.	.	R	.	G	L	A	D	F	M	.	.	T	.	.	.
Past18	.	K	.	.	.	.	V	.	Q	.	Y	.	.	V	E	.	L	R	F	.	.	.	.	.	E	.	.	.	F	.	.	F	.	.	K	.	.	.	.	R	.	G	L	A	D	F	M	.	.	T	.	.	.
Past19	.	K	.	.	L	.	.	.	.	.	Y	.	.	V	E	.	T	R	F	.	.	.	.	.	E	.	.	.	F	.	.	F	.	.	K	.	.	.	.	R	.	.	Q	.	D	F	M	.	T	E	.	.	S
Past21	.	K	.	.	Y	.	D	.	.	.	Y	.	.	V	E	.	A	R	F	.	.	.	.	.	E	.	.	.	F	.	.	.	.	.	K	.	.	.	.	R	.	G	L	A	D	Y	M	.	T	R	.	.	S
Past22	.	K	.	.	.	.	.	.	L	.	Y	.	.	V	E	I	L	R	F	.	.	.	.	.	E	Y	.	.	F	.	.	F	.	.	K	N	.	.	D	.	.	G	L	A	D	Y	M	.	T	K	.	.	S
Past23	.	K	.	.	Y	.	.	.	L	.	Y	.	.	V	E	.	L	R	F	.	.	.	.	.	E	Y	.	.	F	.	.	H	.	.	K	N	.	.	.	R	.	G	L	A	D	F	M	.	T	Q	.	.	S
Past24	.	K	.	.	.	.	V	.	L	.	Y	.	.	V	E	.	A	R	F	.	.	.	.	.	E	.	.	.	F	.	.	H	.	.	K	.	.	.	.	R	.	G	L	A	D	F	M	.	.	T	.	.	.
Past25	.	K	.	.	Y	M	Q	K	L	.	Y	.	.	V	E	.	A	R	F	.	N	N	.	.	K	Y	.	.	F	.	.	.	.	.	K	.	.	K	.	.	.	N	L	.	V	F	M	.	G	R	.	.	.
Past26	.	K	.	.	Y	.	D	.	.	.	Y	.	.	V	E	.	A	R	F	.	.	.	.	.	E	.	.	.	F	.	.	N	.	.	K	.	.	.	.	.	.	G	L	A	D	Y	M	.	R	L	.	.	S
Past28	.	K	.	.	Y	.	D	.	.	.	Y	.	.	V	E	.	A	R	F	.	.	.	.	.	E	.	.	.	F	.	.	.	.	.	K	.	.	.	.	R	.	G	L	A	D	Y	M	.	T	R	.	.	S
Past29	.	K	.	.	.	.	Q	.	F	.	Y	.	.	V	E	.	A	R	F	.	.	.	.	.	E	.	.	.	F	.	.	H	.	.	K	N	.	.	.	.	.	G	Q	S	D	L	M	.	R	P	.	.	.
Past31	.	K	.	.	Y	.	D	.	L	.	Y	.	.	V	E	.	L	T	F	.	.	.	.	.	E	Y	.	.	F	.	.	H	.	.	K	N	.	.	I	.	.	G	L	A	D	Y	M	.	R	K	.	.	.
Past32	.	K	.	.	Y	.	D	.	H	.	Y	.	.	V	E	H	A	R	F	.	.	.	.	.	E	.	.	.	F	.	.	.	.	.	K	.	.	.	.	R	.	G	L	A	D	Y	M	.	T	R	.	.	.
Past33	.	K	.	.	.	.	V	.	L	.	Y	.	.	V	E	.	L	R	F	.	.	.	.	.	E	.	.	.	F	.	.	H	.	.	K	N	.	.	I	.	.	G	L	A	D	Y	M	.	R	K	.	.	S
Past34	.	K	.	.	Y	.	D	.	.	.	Y	.	.	V	E	.	A	R	F	.	.	.	.	.	E	.	.	.	F	.	.	.	.	.	K	.	.	.	.	R	.	.	Q	.	D	F	M	.	T	Q	.	.	S
Past35	.	K	.	.	Y	.	.	.	L	.	Y	.	.	V	E	.	T	R	F	.	.	.	.	.	E	.	.	.	F	.	.	H	.	.	K	.	.	.	.	R	.	G	L	A	V	Y	M	.	E	.	.	.	.
Past36	.	K	.	.	.	.	V	.	Q	.	Y	.	.	V	E	.	A	R	F	.	.	.	.	.	E	.	.	.	F	.	.	.	.	.	K	.	.	.	.	R	.	G	L	A	D	F	M	.	.	T	.	.	.
Past39	.	K	.	.	L	L	V	K	.	.	Y	.	.	.	Q	L	.	H	F	.	.	.	.	.	.	.	.	.	.	.	.	F	.	.	K	V	.	.	N	.	D	.	N	.	V	.	M	.	R	R	.	A	E
Past40	.	K	.	.	L	.	.	.	.	.	Y	.	.	V	E	.	T	R	F	.	.	.	.	.	E	.	.	.	F	.	.	.	.	.	K	.	.	.	.	R	.	G	L	A	D	F	M	.	T	R	.	.	.
Past41	.	K	.	.	.	L	.	.	H	.	Y	.	.	V	E	I	L	R	F	.	.	.	.	.	E	Y	.	.	F	.	.	F	.	.	K	.	.	.	I	.	.	G	L	A	D	Y	M	.	T	R	.	.	.
Past42	.	K	.	.	.	L	Q	.	.	.	Y	.	.	L	E	D	.	R	F	.	.	.	.	.	K	.	.	.	F	.	.	A	.	D	K	W	.	Q	D	.	.	.	.	E	A	R	M	.	Y	T	.	A	Q
Past44	.	K	.	.	Y	.	D	.	.	.	Y	.	.	V	E	.	A	R	F	.	.	.	.	.	E	.	.	.	F	.	.	.	.	.	K	.	.	.	.	R	.	G	L	A	D	Y	M	.	T	R	.	.	S

Additional file 4 Amino acid alignment of 159 nucleotide sites of MHC class II β exon 2 from seven basal passerine species. Amino acid residues distinguishing Meliphagidae, Pardalotidae and Climacteridae are highlighted in grey
